# Current status of surveillance for Barrett's esophagus in Japan and the West

**DOI:** 10.1002/deo2.94

**Published:** 2022-02-13

**Authors:** Tomoyuki Koike, Masahiro Saito, Yuki Ohara, Waku Hatta, Atsushi Masamune

**Affiliations:** ^1^ Division of Gastroenterology Tohoku University Graduate School of Medicine Miyagi Japan

**Keywords:** Barrett's esophagus, Barrett's adenocarcinoma, surveillance

## Abstract

Prospective studies in western countries have shown that the obvious risk factors for Barrett's esophageal cancer are male sex, smoking habit, a longer length of Barrett's esophagus, and low‐grade dysplasia. However, few reports have prospectively examined risk factors for adenocarcinoma development from Barrett's esophagus in Japan. In the West, where adenocarcinoma is common among esophageal cancer, endoscopic surveillance of Barrett's esophagus every 2–5 years is recommended for early detection of adenocarcinoma. However, there is no established surveillance method in Japan. In recent years, the incidence of adenocarcinoma from long‐segment Barrett's esophagus and short‐segment Barrett's esophagus longer than 2 cm in Japan has been reported to be similar to the West. For surveillance of adenocarcinoma arising from Barrett's esophagus, recognizing the characteristics of superficial adenocarcinoma and carefully observing the entire Barrett's esophagus are needed. It has been reported that representative characteristics of Barrett's adenocarcinoma are a reddish area or a lesion located on the anterior to the right sidewall. It is necessary to establish surveillance methods for Barrett's esophagus sooner in Japan.

## INTRODUCTION

An increase in Barrett's adenocarcinoma has been reported in Japan as well as in western countries.^1^, ^2^, ^3^, ^4^, ^5^ Barrett's esophagus is considered to be a complication of gastroesophageal reflux disease (GERD). Early detection of Barrett's adenocarcinoma is important since superficial Barrett's adenocarcinoma after endoscopic treatment has a favorable prognosis.^6^, ^7^ This article will review the risk of carcinogenesis and the current status of surveillance of Barrett's esophagus in Japan compared with the West.

## ETIOLOGY OF BARRETT'S ESOPHAGUS

The development of Barrett's esophagus is thought to be related to the reflux of gastric acid and bile into the esophagus and the presence of mucosal damage associated with reflux esophagitis. In fact, studies using esophageal pH monitoring have reported that acid exposure time in the esophagus is associated with the presence and length of Barrett's esophagus.^8^ Furthermore, bilirubin exposure time in the esophagus is associated with the presence and length of Barrett's esophagus.^9^, ^10^ It has also been shown that the combination of gastric and bile acids further increases the risk of developing Barrett's esophagus.^9^, ^10^

## DEFINITION AND DIAGNOSIS OF BARRETT'S ESOPHAGUS

**FIGURE 1:**
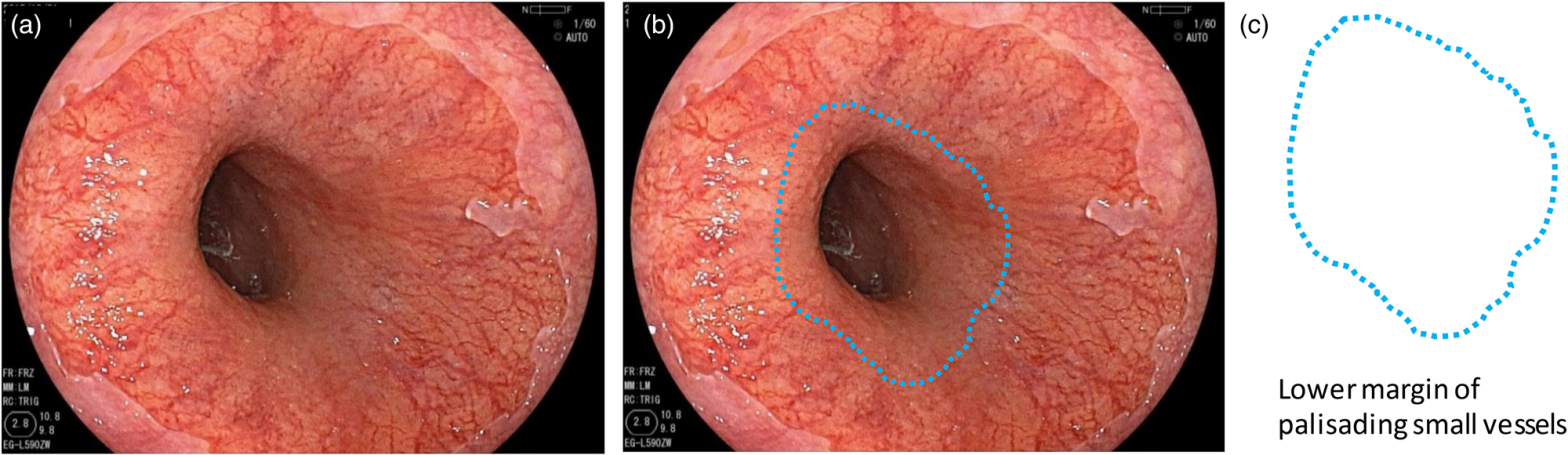
Definition of the esophagogastric junction (EGJ) according to the Japanese Classification of Esophageal Cancer. Endoscopic Findings. Lower margin of palisading small vessels. If the palisading small vessels are unclear, the oral margin of the longitudinal folds of the greater curvature of the stomach is defined as the EGJ. Modified from Japanese Classification of Esophageal Cancer

**FIGURE 2:**
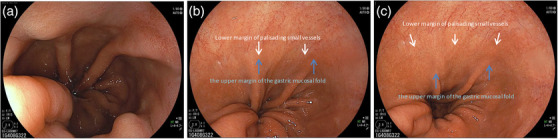
Relationship between the oral edge of the gastric mucosal folds and the lower edge of the palisading small vessels. The oral edge of the gastric mucosal fold changes easily depending on airflow and the degree of inspiration. (a) When airflow is reduced, the upper margin of the gastric mucosal folds moves more easily to the oral side. The palisading small vessels also become less visible. (b) By adjusting the airflow and inspiration, the upper margin of the gastric mucosal fold coincides with the lower margin of the palisading small vessels. (c) As airflow increases, the upper margin of the gastric mucosal folds moves more easily to the anal side

Barrett's esophagus is defined as a condition in which the mucosa of the lower esophagus has been replaced by a continuous columnar epithelium from the stomach. To endoscopically diagnose Barrett's esophagus, the esophagogastric junction (EGJ) must be identified. According to the Japanese Classification of Esophageal Cancer edited by the Japan Esophageal Society, the EGJ is defined as the lower margin of palisading small vessels in the lower esophagus on endoscopy, or if the palisading small vessels are unclear, the oral margin of the longitudinal folds of the greater curvature of the stomach is defined as the EGJ (Figure 1).^11^ On the other hand, in western countries, “the upper margin of the gastric mucosal fold” is mainly used as the definition of EGJ, but the oral edge of the gastric mucosal fold changes easily depending on airflow and the degree of inspiration (Figure 2).

**FIGURE 3:**
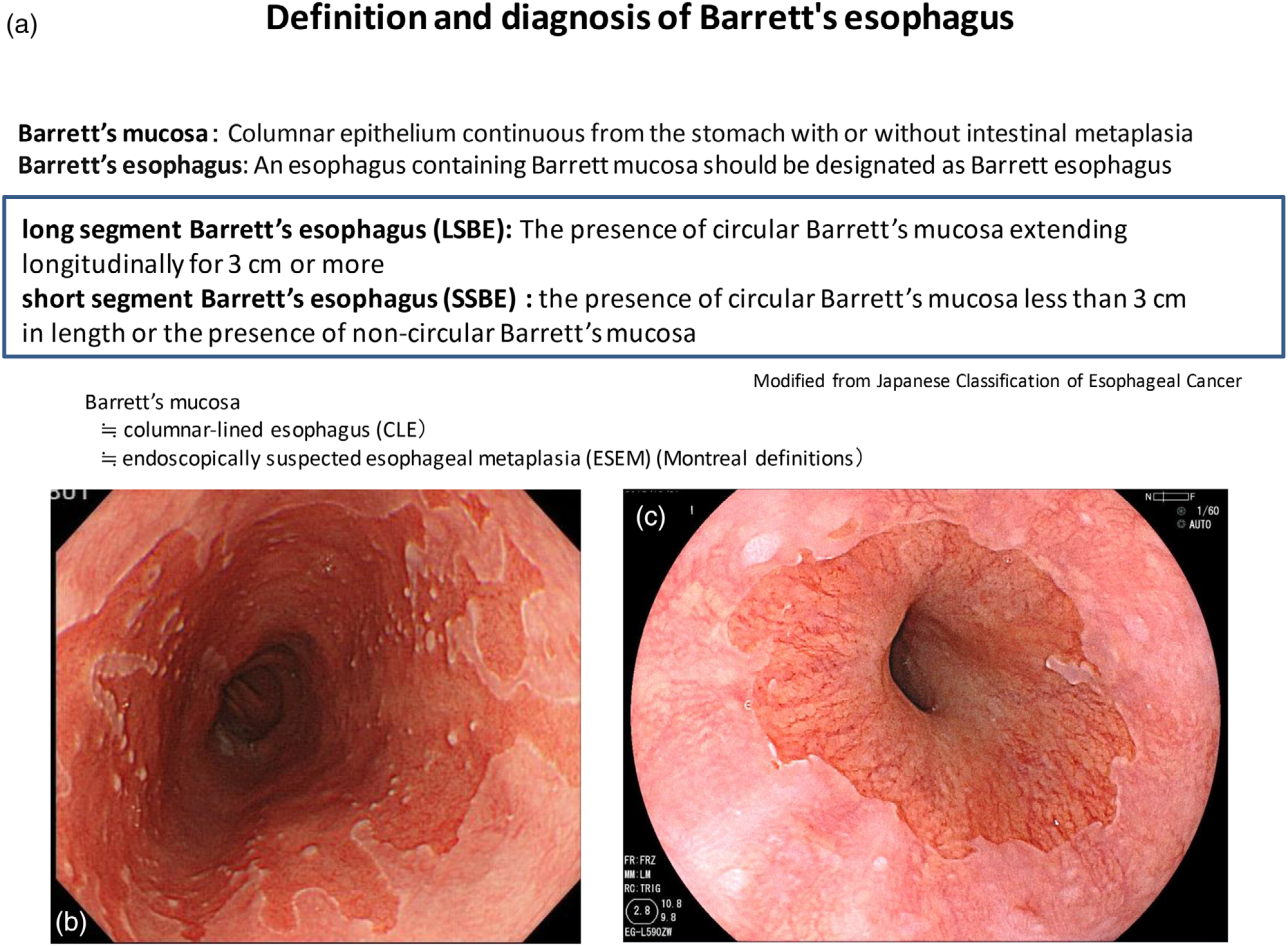
Definition and diagnosis of Barrett's esophagus according to the Japanese Classification of Esophageal Cancer. (a) Definition and diagnosis of Barrett's esophagus. (b) Long‐segment Barrett's esophagus (LSBE). (c) Short‐segment Barrett's esophagus (SSBE)

Once the EGJ is determined, Barrett's esophagus can easily be diagnosed, but it is important to note the difference in definitions between Japan and the West. In most western countries, Barrett's esophagus is defined as the presence of a specialized columnar epithelium with intestinal metaplasia (IM) with goblet cells because of the increased risk of carcinogenesis.^12^, ^13^, ^14^ According to the American College of Gastroenterology (ACG) guidelines, Barrett's esophagus is diagnosed by the presence of IM on biopsy in addition to the presence of columnar epithelium of at least 1 cm in the esophagus (Table 1).^15^ On the other hand, in Japan, the definition of Barrett's mucosa by the Japan Esophageal Society is a columnar epithelium continuous from the stomach with or without IM, and an esophagus containing Barrett's mucosa should be designated as Barrett's esophagus. The definition of long‐segment Barrett's esophagus (LSBE) is the presence of circular Barrett's mucosa extending longitudinally for 3 cm or more, and the presence of circular Barrett mucosa less than 3 cm in length or the presence of non‐circular Barrett's mucosa is designated as short‐segment Barrett's esophagus (SSBE) (Figure 3). On the other hand, in western countries, Barrett's esophagus with a maximum length of 3 cm is defined as an LSBE.

**TABLE 1:** Diagnostic criteria for Barrett's esophagus in different countries

**Guidelines**	**Length criteria**	**Histology criteria**
AGA	Any extent	Intestinal metaplasia
ASGE	None	Intestinal metaplasia
BSG	≥ 1 cm	Columnar epithelium
Australia	Any extent	Intestinal metaplasia
ACG	≥ 1 cm	Intestinal metaplasia
ESGE	≥ 1 cm	Intestinal metaplasia
APAGE	≥ 1 cm	Columnar epithelium

Abbreviations: ACG, American College of Gastroenterology; AGA, American Gastroenterological Association; APAGE, Asian Pacific Association of Gastroenterology; ASGE, American Society for Gastrointestinal Endoscopy; BSG, British Society of Gastroenterology; ESGE, European Society for Gastrointestinal Endoscopy; IM, intestinal metaplasia.

## RISK FACTORS FOR BARRETT'S ADENOCARCINOMA

In Japan, obesity, hiatal hernia, smoking, and being male have been reported as risk factors for the development of EGJ cancer, including Barrett's adenocarcinoma.^16^ However, there are no data prospectively examining risk factors for developing adenocarcinoma from Barrett's esophagus. The incidence of Barrett's esophageal cancer is higher in western countries than in Japan.^17^ A study of racial differences in the United States clearly showed a higher frequency in non‐Hispanic Caucasians and a lower frequency in Asians.^18^, ^19^ In addition, males have a higher risk of carcinogenesis. The male to female ratio is reported to be about 9:1 in the United States.^17^, ^20^, ^21^, ^22^, ^23^ Aging has also been recognized as a risk factor for Barrett's esophageal cancer.^24^, ^25^

Although there are many reports on the causal relationship between obesity and Barrett's adenocarcinoma,^26^, ^27^, ^28^, ^29^ some meta‐analyses have shown no association between Body Mass Index (BMI) and Barrett's esophagus carcinogenesis.^30^ On the other hand, a meta‐analysis reported that central obesity is a risk factor for carcinogenesis.^31^

Many reports show a causal relationship between smoking and Barrett's adenocarcinoma.^24^ Smokers have about twice the risk of carcinogenesis compared to non‐smokers.^32^, ^33^ There are many reports that alcohol consumption is not associated with the risk of Barrett's adenocarcinoma.^32^

It has been reported from the West that the risk of cancer increases by an odds ratio of 1.11 for every 1 cm increase in the length of Barrett's esophagus.^34^ The presence of low‐grade dysplasia (LGD) in Barrett's esophagus has also been shown to increase the risk of carcinogenesis.^30^

In a report that scored the risk of carcinogenesis, 9 points were given to males, 5 points to the smoking habit, 1 point per 1 cm of Barrett's esophagus length, and 11 points for confirmed LGD, with a total of 20 points or more being considered high risk for carcinogenesis (annual carcinogenesis rate of 2.1%) and 10 points or less being considered low risk (annual carcinogenesis rate of 0.13%).^35^

## INCIDENCE OF ADENOCARCINOMA FROM BARRETT'S ESOPHAGUS

The incidence of carcinogenesis from Barrett's esophagus in the West is 0.3%–0.6% per year.^36^, ^37^, ^38^ A meta‐analysis comparing the incidence of adenocarcinoma in SSBE and LSBE reported that the annual rate of carcinogenesis, including high‐grade dysplasia, was 0.76% in LSBE compared with 0.24% in SSBE.^39^ However, in the report of a multicenter prospective cohort study of LSBE conducted by the Japanese Society of Gastrointestinal Endoscopy, the incidence of adenocarcinoma from LSBE followed up for more than 1 year was 1.2% per year.^40^ Recently, a longer‐term report on Barrett's esophagus longer than 2 cm reported an annual incidence of adenocarcinoma of 0.47%.^41^ Therefore, the incidence of carcinoma from LSBE and SSBE longer than 2 cm in Japan is comparable to that from Barrett's esophagus in the West. However, it cannot be denied that the differences in the definition of Barrett's esophagus, including some selection biases and histopathological diagnosis between Japan and the West^11^ may influence this.

## BARRETT'S ESOPHAGUS AS A TARGET FOR SURVEILLANCE

According to the definition of Barrett's esophagus in Japan, in which the “lower end of the palisading small vessels” is defined as EGJ with or without IM, the incidence of Barrett's esophagus including SSBE is high, and the highest reported was 85.9% of endoscopic examinations.^42^, ^43^, ^44^, ^45^ Therefore, it is impossible to consider that all Barrett's esophagus, according to the Japanese definition, increases the risk of cancer. In other words, it is important to determine which Barrett's esophagus may lead to cancer. The diagnostic criteria for Barrett's esophagus in the British Society of Gastroenterology (BSG) guidelines state that proof of IM is not necessary, but only IM‐positive Barrett's esophagus cases require surveillance (Tables 1 and 2).^46^

**TABLE 2:** Guidelines for Barrett's esophagus surveillance in different countries

**Guidelines**	**Length‐based criteria**	**Interval**
AGA	No	3–5 years
ASGE	No	3–5 years
BSG	<3 cm with IM	3–5 years
	≥3 cm with IM	2–3 years
Australia	<3 cm	3–5 years
	>3 cm	2–3 years
ACG	No	3–5 years
ESGE	≥1 cm and <3 cm	5 years
	≥3 cm and <10 cm	3 years
	≥10 cm	Expert center management
APAGE	No	3–5 years

Abbreviations: ACG, American College of Gastroenterology; AGA, American Gastroenterological Association; APAGE, Asian Pacific Association of Gastroenterology; ASGE, American Society for Gastrointestinal Endoscopy; BSG, British Society of Gastroenterology; ESGE, European Society for Gastrointestinal Endoscopy; IM, intestinal metaplasia.

The risk of carcinogenesis in Barrett's esophagus has been strongly related to the length of Barrett's esophagus,^38^ and the annual rate of carcinogenesis from LSBE in Japan is as high as 1.2%.^40^ However, LSBE accounts for less than 1% of Barrett's esophagus in Japan,^42^ so the necessity for surveillance of the entire Barrett's esophagus has not yet been determined in Japan. The guidelines by the Asian Pacific Association of Gastroenterology state that, at present, there is no proven benefit from endoscopic surveillance of Barrett's esophagus in the absence of dysplasia.^47^ In many West and Asian countries' guidelines, ultra‐SSBE (USSBE) of less than 1 cm is not included in the diagnosis of Barrett's esophagus or surveillance (Tables 1 and 2).^14^, ^47^ The carcinogenic potential of USSBE is regarded as negligible, with a recent study from the United States noting no cancer development in any of 167 patients with USSBE during a median follow‐up of 5.9 years.^48^ Therefore, stratification of Barrett's esophagus according to the length and other factors to narrow down the target population for surveillance would be an important issue in the future.

The risk of cancer in the shorter forms of Barrett's esophagus (USSBE + SSBE) in Japanese populations has been largely unknown. However, a recent retrospective cohort study in Japan reported that although the prevalence of USSBE is high (36.4%), the incidence of adenocarcinoma in USSBE is very low (0.0068% per year).^49^ In addition, the aforementioned annual incidence of 0.47% of adenocarcinoma from SSBE longer than 2 cm, in Japan,^41^ may be an important indicator for future stratification of Barrett's esophagus.

## SURVEILLANCE METHODS OF BARRETT'S ESOPHAGUS

In the West, endoscopic surveillance every 2–5 years is recommended for Barrett's esophagus patients for early detection of adenocarcinoma (Table 2).^14^, ^47^ However, there are no prospective randomized controlled trials that have shown efficacy in improving the mortality of Barrett's esophagus patients.^50^ As for the actual method of endoscopic surveillance, random four‐quadrant biopsies at 2 cm intervals in patients without dysplasia and 1 cm intervals in patients with prior dysplasia (Seattle protocol) are recommended in the West, as described in the ACG guidelines.^15^ However, this biopsy protocol is time‐consuming, risks sampling error, and is hampered by low patient compliance. In addition, endoscopic resection is recommended in the presence of mucosal irregularities. Barrett's esophagus without dysplasia has a recommended surveillance period of every 3–5 years (Table 2), and endoscopic treatment is recommended for Barrett's esophagus with dysplasia. Histopathological diagnosis of Barrett's esophagus with dysplasia is strongly recommended to be made by two pathologists, including at least one who specializes in gastrointestinal pathology.

On the other hand, there are many reports of observation of Barrett's mucosal pattern by magnifying endoscopy with image‐enhanced endoscopy (IEE), such as acetic acid and narrow‐band imaging (NBI) as an effective alternative to random biopsy.^47^ In addition, the usefulness of linked color imaging (LCI) has also been reported recently.^51^, ^52^ In Japan, surveillance methods for Barrett's esophagus have not been established, but unlike in the West, random biopsies are rarely performed, and targeted biopsies of suspected lesions are commonly done.

BSG guidelines and other guidelines suggest changing the interval of surveillance according to the length of Barrett's esophagus (Table 2).^46^, ^53^ Furthermore, although risk assessment based on the presence or absence of dysplasia has been studied, there is insufficient evidence in the West.

Sharma et al. reported in an international randomized crossover trial that targeted biopsies under NBI had a similar detection rate to IM and fewer biopsies than the Seattle protocol under white light, and regular mucosal surface patterns under NBI did not detect high‐grade dysplasia or cancer. Therefore, it is suggested to avoid biopsies from these areas.^54^ Furthermore, international standards for NBI endoscopy diagnoses have been developed, and their usefulness has been demonstrated.^55^ In addition, the targeted biopsy combined with the acetic acid method has been developed,^56^ as international endoscopic diagnostic criteria.^57^

In a meta‐analysis conducted by the American Society for Gastrointestinal Endoscopy, it was reported that targeted biopsies with acetic acid, NBI, and endoscope‐based confocal laser endomicroscopy are diagnostic methods that can replace random biopsy.^58^ Therefore, targeted biopsies may replace random biopsies in western countries in the future.

## CASE PRESENTATIONS: PATIENTS WITH ADENOCARCINOMA DETECTED DURING BARRETT'S ESOPHAGUS SURVEILLANCE

We present two cases of Barrett's adenocarcinoma detected during surveillance of Barrett's esophagus.

### Case 1 (Figure 4)

The index endoscopy revealed reflux esophagitis and LSBE were observed, and annual surveillance by endoscopy was started. Three years after the index endoscopy, reflux esophagitis and GERD symptoms worsened, and maintenance therapy with proton pump inhibitor (PPI) was started. 12 years after the index endoscopy, the patient was still taking PPI, but an irregular depressed surface with a clear demarcation line was observed at the oral side of the LSBE, and the diagnosis of adenocarcinoma was suspected. Well‐differentiated type adenocarcinoma was suspected by biopsy, and endoscopic submucosal dissection (ESD) was performed. The histopathological diagnosis was adenocarcinoma in Barrett's esophagus, macroscopic type 0‐IIc, tumor size 8 × 7 mm, well‐differentiated type, pT1a‐DMM, ly(‐), v(‐), pHM0, pVM0.

**FIGURE 4:**
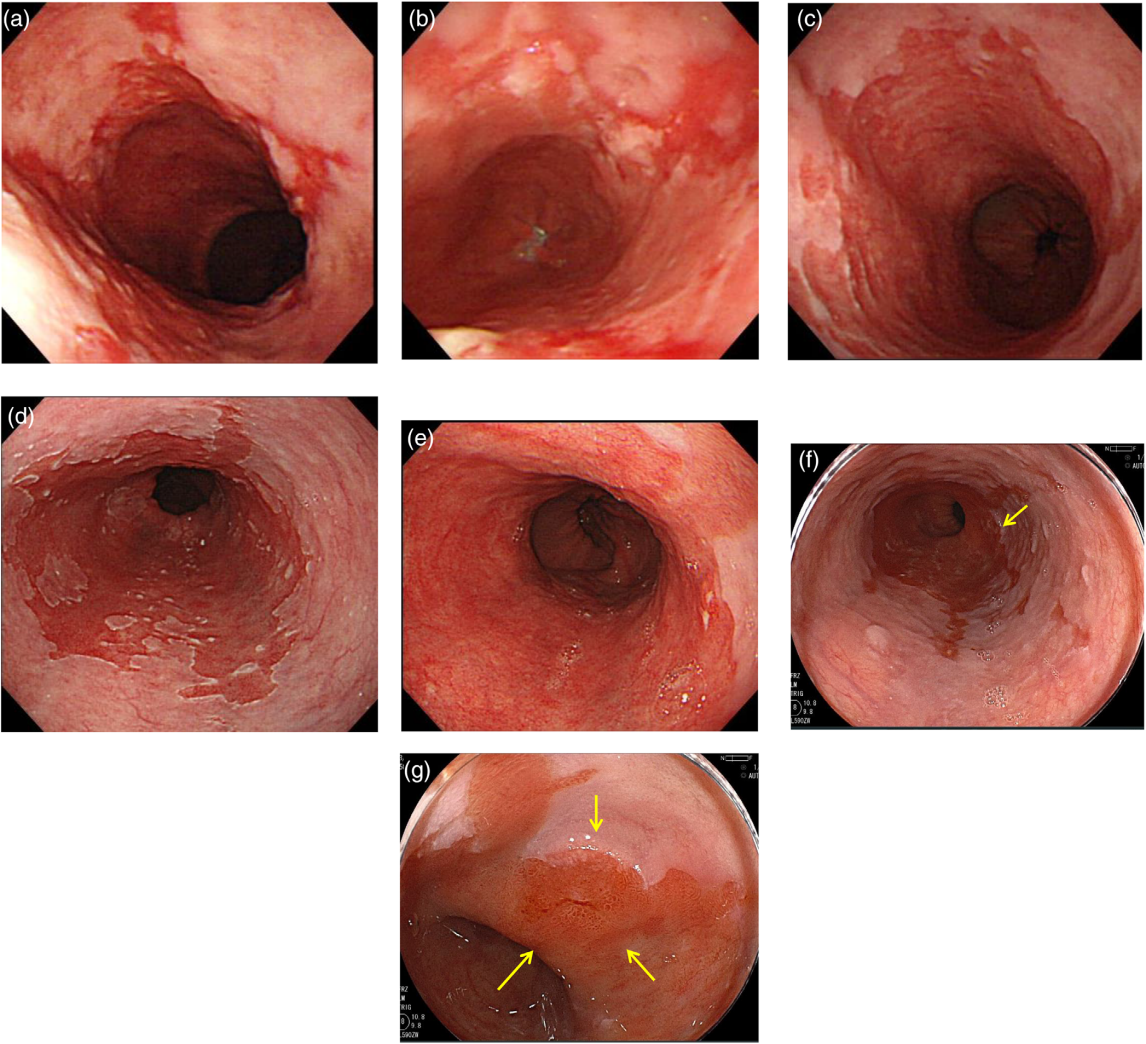
Case 1: Patient with adenocarcinoma detected during long‐segment Barrett's esophagus (LSBE) surveillance. The index endoscopy revealed reflux esophagitis and LSBE, and annual surveillance by endoscopy was started. 12 years after the index endoscopy, an irregular depressed surface with a clear demarcation line was observed at the oral side of the LSBE, and the diagnosis of adenocarcinoma was suspected (yellow arrows). A biopsy revealed adenocarcinoma. Endoscopic submucosal dissection (ESD) was performed. The histopathological diagnosis was adenocarcinoma in Barrett's esophagus, macroscopic type 0‐IIc, tumor size 8 × 7 mm, well‐differentiated, pT1a‐DMM, ly(‐), v(‐), pHM0, and pVM0. (a) The index endoscopy revealed reflux esophagitis and LSBE. (b) Follow‐ups were conducted for the patient, by annual surveillance endoscopy. Three years after the index endoscopy. (c) Six years after the index endoscopy. (d) Ten years after the index endoscopy. (e) Eleven years after the index endoscopy. (f, g) Barrett's adenocarcinoma was detected 12 years after the index endoscopy

### Case 2 (Figure 5)

The index endoscopy revealed SSBE, and annual surveillance through endoscopy was started. Six years after the index endoscopy, an irregular protruded lesion was observed at the oral side of the SSBE, and the diagnosis of adenocarcinoma was suspected. A biopsy revealed adenocarcinoma, and ESD was performed. The histopathology showed adenocarcinoma in Barrett's esophagus, macroscopic type 0‐IIa, tumor size 8 × 7 mm, well‐differentiated type, pT1a‐DMM, ly(‐), v(‐), pHM0, pVM0.

**FIGURE 5:**
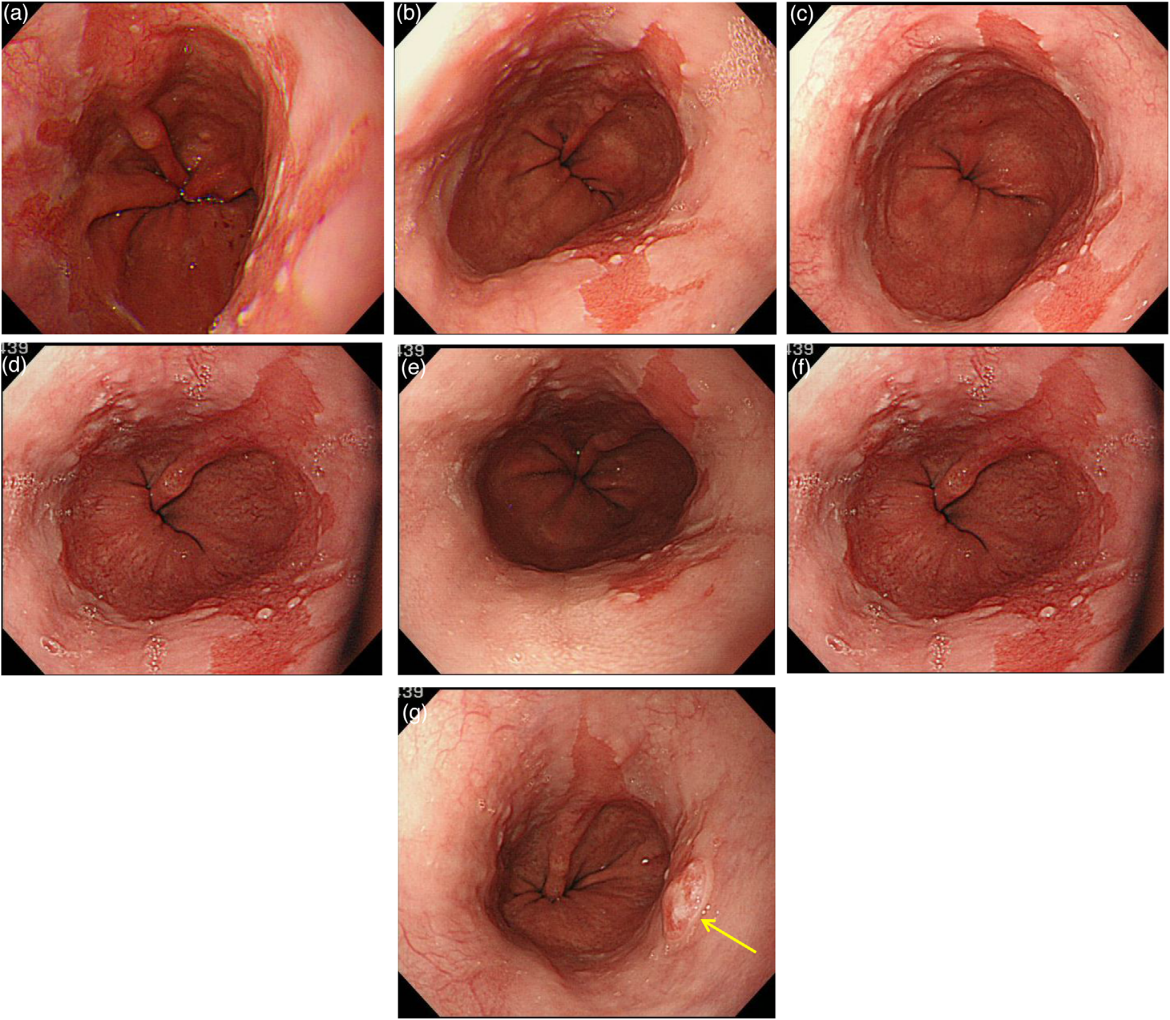
Case 2: Patient with adenocarcinoma detected during short‐segment Barrett's esophagus (SSBE) surveillance. The index endoscopy revealed SSBE, and annual surveillance by endoscopy was started. Six years after the index endoscopy, an irregular protruded lesion was observed at the oral side of the SSBE, and the diagnosis of adenocarcinoma was suspected (yellow arrow). A biopsy revealed adenocarcinoma. ESD was performed. The histopathology showed adenocarcinoma in Barrett's esophagus, macroscopic type 0‐IIa, tumor size 8 × 7 mm,well‐differentiated, pT1a‐DMM, ly(‐), v(‐), pHM0, and pVM0. (a) The index endoscopy revealed SSBE. (b) Follow‐ups were conducted for the patient, by annual surveillance endoscopy. One year after the index endoscopy. (c) Two years after the index endoscopy. (d) Three years after the index endoscopy. (e) Four years after the index endoscopy. (f) Five years after the index endoscopy. (g) Barrett's adenocarcinoma was detected 6 years after the index endoscopy

## ENDOSCOPIC CHARACTERISTICS OF SUPERFICIAL BARRETT'S ADENOCARCINOMA

**FIGURE 6:**
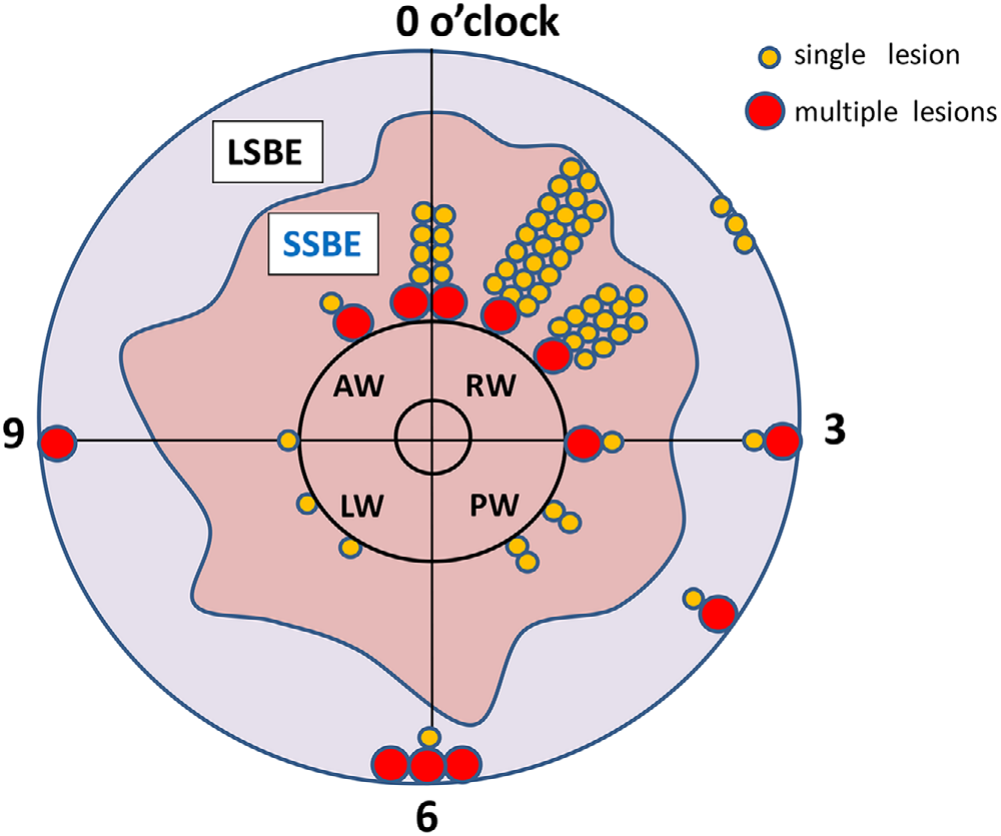
Location of superficial Barrett's adenocarcinoma. Most of the lesions are found in the 0–3 o'clock direction. A high percentage of lesions are located in the 6 o'clock direction in LSBE cases, and there are many cases of multiple lesions in LSBE LSBE, long‐segment Barrett's esophagus; SSBE, short‐segment Barrett's esophagus; AW, anterior wall; PW, posterior wall, RW; right wall, LW; left wall. Modified^61^

To detect Barrett's adenocarcinoma in surveillance, good knowledge of endoscopic characteristics is essential. It has been reported that representative characteristics of Barrett's adenocarcinoma are a reddish area or a lesion located anterior to the right sidewall.^59^, ^60^, ^61^ In our department, about 90% of the superficial Barrett's adenocarcinoma observed also showed reddishness. Most of the lesions were found in the 0–3 o'clock direction, from the anterior to the right wall (Figure 6).^62^ Thus, it is important to focus on the reddish area located on the anterior to the right wall in Barrett's mucosa to detect adenocarcinoma. In addition, although the number of cases is less, we have seen a high percentage of lesions that are located in the 6 o'clock direction in LSBE cases, with many cases of multiple lesions in LSBE (Figure 6).^61^ Therefore, in clinical practice when a single lesion is found in the LSBE, it is important to be aware of lesions in other areas.

## CONCLUSIONS

Recognizing the characteristics of superficial Barrett's adenocarcinoma and careful observation of the entire Barrett's esophagus are needed to monitor adenocarcinoma arising from Barrett's esophagus. IEE such as the acetic acid, NBI, and LCI methods could be useful for surveillance. However, no multicenter, prospective study has been reported in Japan. The surveillance method for Barrett's adenocarcinoma needs to be established in Japan in the near future.

## CONFLICT OF INTEREST

The authors have no conflicts of interest to be declared with regard to this study. WH is an associate editor of DEN Open.

## FUNDING INFORMATION

None.
